# Survival of patients with deficient mismatch repair metastatic colorectal cancer in the pre-immunotherapy era

**DOI:** 10.1038/s41416-020-01076-0

**Published:** 2020-10-13

**Authors:** G. Emerens Wensink, Marloes A. G. Elferink, Anne M. May, Linda Mol, Patricia A. H. Hamers, Sandra D. Bakker, Geert-Jan Creemers, Jan Willem B. de Groot, Gerty J. de Klerk, Brigitte C. M. Haberkorn, Annebeth W. Haringhuizen, Ronald Hoekstra, J. Cornelis B. Hunting, Emile D. Kerver, Danielle Mathijssen-van Stein, Marco B. Polée, Johannes F. M. Pruijt, Patricia Quarles van Ufford-Mannesse, Sandra Radema, Ronald C. Rietbroek, Lieke H. J. Simkens, Bea C. Tanis, Daan ten Bokkel Huinink, Manuel L. R. Tjin-A-Ton, Cathrien S. Tromp-van Driel, Monique M. Troost, Agnes J. van de Wouw, Franchette W. P. J. van den Berkmortel, Anke J. M. van der Pas, Ankie M. T. van der Velden, Marjan A. van Dijk, Joyce M. van Dodewaard-de Jong, Edith B. van Druten, Theo van Voorthuizen, Gerrit Jan Veldhuis, Henk M. W. Verheul, Hanneke J. H. M. J. Vestjens, Jeroen Vincent, Onno W. Kranenburg, Cornelis J. A. Punt, Geraldine R. Vink, Jeanine M. L. Roodhart, Miriam Koopman

**Affiliations:** 1 Department of Medical Oncology, University Medical Center Utrecht, Utrecht University, Universiteitsweg 100, 3584CG Utrecht, The Netherlands; 2 Department of Research, Netherlands Comprehensive Cancer Organisation, Postbus 19079, 3501DB Utrecht, The Netherlands; 3 Julius Center for Health Sciences and Primary Care, University Medical Center Utrecht, Utrecht University, P.O. Box 85500, 3508GA Utrecht, The Netherlands; 4 Department of Medical Oncology, Zaans Medical Center, Postbus 210, 1500EE Zaandam, The Netherlands; 5 Department of Medical Oncology, Catharina Hospital, Postbus 1350, 5602ZA Eindhoven, The Netherlands; 6 Department of Medical Oncology, Isala Hospital, Dokter van Heesweg 2, 8025AB Zwolle, The Netherlands; 7 Department of Medical Oncology, Spaarne Gasthuis, Spaarnepoort 1, 2134TM Hoofddorp, The Netherlands; 8 Department of Medical Oncology, Maasstad Hospital, Postbus 9100, 3007AC Rotterdam, The Netherlands; 9 Department of Medical Oncology, Hospital Gelderse Vallei, Willy Brandtlaan 10, 6716RP Ede, The Netherlands; 10 Department of Medical Oncology, Ziekenhuisgroep Twente, Postbus 7600, 7600SZ Almelo, The Netherlands; 11 Department of Medical Oncology, St. Antonius Hospital, Postbus 2500, 3430EM Nieuwegein, The Netherlands; 12 Department of Medical Oncology, OLVG, Oosterpark 9, 1091AC Amsterdam, The Netherlands; 13 Department of Medical Oncology, Franciscus Gasthuis & Vlietland, Vlietlandplein, 3118JH Schiedam, The Netherlands; 14 Department of Medical Oncology, Medical Center Leeuwarden, Postbus 888, 8901BR Leeuwarden, The Netherlands; 15 Department of Medical Oncology, Jeroen Bosch Hospital, Postbus 90153, 5200ME ‘s-Hertogenbosch, The Netherlands; 16 Department of Medical Oncology, HagaZiekenhuis, Els Borst-Eilersplein 275, 2545AA den Haag, The Netherlands; 17 Department of Medical Oncology, Radboud University Medical Center, Postbus 9101, 6500HB Nijmegen, the Netherlands; 18 Department of Medical Oncology, Rode Kruis Hospital, Postbus 1074, 1940EB Beverwijk, The Netherlands; 19 Department of Medical Oncology, Maxima Medical Center, Postbus 90052, 5600PD Eindhoven, The Netherlands; 20 Department of Medical Oncology, Groene Hart Hospital, Bleulandweg 10, 2803HH Gouda, The Netherlands; 21 Department of Medical Oncology, Diakonessenhuis Utrecht, Postbus 80250, 3508TG Utrecht, The Netherlands; 22 Department of Medical Oncology, Hospital Rivierenland, President Kennedylaan 1, 4002WP Tiel, The Netherlands; 23 Department of Medical Oncology, Gelre Hospital, Postbus 9014, 7300DS Apeldoorn, The Netherlands; 24 Department of Medical Oncology, Bravis Hospital Bergen op Zoom, Postbus 999, 4700AZ Roosendaal, The Netherlands; 25 Department of Medical Oncology, VieCuri Medical Center, Postbus 1926, 5900BX Venlo, The Netherlands; 26 Department of Medical Oncology, Zuyderland Medical Center Heerlen, Postbus 5500, 6130MB Sittard-Geleen, The Netherlands; 27 Department of Medical Oncology, LangeLand Hospital, Postbus 3015, 2700KJ Zoetermeer, The Netherlands; 28 Department of Medical Oncology, Tergooi, Van Riebeeckweg 212, 1213XZ Hilversum, The Netherlands; 29 Department of Medical Oncology, ZorgSaam Hospital, Wielingenlaan 2, 4535PA Terneuzen, The Netherlands; 30 Department of Medical Oncology, Meander Medical Center, Postbus 1502, 3800BM Amersfoort, The Netherlands; 31 Department of Medical Oncology, Reinier de Graaf Gasthuis, Postbus 5011, 2600GA Delft, The Netherlands; 32 Department of Medical Oncology, Hospital Rijnstate, Wagnerlaan 55, 6815AD Arnhem, The Netherlands; 33 Department of Medical Oncology, Antonius Hospital Sneek, Postbus 20000, 8600BA Sneek, The Netherlands; 34 Department of Medical Oncology, Amsterdam University Medical Center, VU Medical Center, P.O. Box 7057, 1007MB Amsterdam, The Netherlands; 35 Department of Medical Oncology, Elkerliek Hospital, Postbus 98, 5700AB Helmond, The Netherlands; 36 Department of Surgical Oncology, University Medical Center Utrecht, Utrecht University, Postbus 98, 5700AB Utrecht, The Netherlands; 37 Utrecht Platform for Organoid Technology, University Medical Center Utrecht, Utrecht University, Postbus 98, 5700AB Utrecht, The Netherlands; 38 Department of Medical Oncology, Amsterdam University Medical Centers, University of Amsterdam, Postbus 22660, Amsterdam, The Netherlands

**Keywords:** Colorectal cancer, Cancer therapy

## Abstract

**Background:**

Metastatic colorectal cancer patients with deficient mismatch repair (dMMR mCRC) benefit from immunotherapy. Interpretation of the single-arm immunotherapy trials is complicated by insignificant survival data during systemic non-immunotherapy. We present survival data on a large, comprehensive cohort of dMMR mCRC patients, treated with or without systemic non-immunotherapy.

**Methods:**

Two hundred and eighty-one dMMR mCRC patients (*n* = 54 from three prospective Phase 3 CAIRO trials; *n* = 227 from the Netherlands Cancer Registry). Overall survival was analysed from diagnosis of mCRC (OS), from initiation of first-line (OS1) and second-line (OS2) systemic treatment. Cox regression analysis examined prognostic factors. As comparison for OS 2746 MMR proficient mCRC patients were identified.

**Results:**

Of 281 dMMR patients, 62% received first-line and 26% second-line treatment. Median OS was 16.0 months (13.8–19.6) with antitumour therapy and 2.5 months (1.8–3.5) in untreated patients. OS1 was 12.8 months (10.7–15.2) and OS2 6.2 months (5.4–8.9) in treated dMMR patients. Treated dMMR patients had a 7.6-month shorter median OS than pMMR patients.

**Conclusion:**

Available data from immunotherapy trials lack a control arm with standard systemic treatment. Given the poor outcome compared to the immunotherapy results, our data strongly suggest a survival benefit of immunotherapy in dMMR mCRC patients.

## Background

Approximately 5% of metastatic colorectal cancer (mCRC) patients have a tumour with deficient DNA mismatch repair (dMMR), also referred to as microsatellite instability.^1^ dMMR arises through germline mutations or epigenetic methylation and inactivation of the MMR pathway, resulting in insertions or deletions in tandem repetitive sequences in DNA, a hypermutated genome, and a strong immune infiltrate.^2^ Sporadic dMMR tumours frequently harbour a *BRAFV600E* mutation and sporadic dMMR patients have a worse prognosis compared to Lynch syndrome patients.^2,3^

A dMMR tumour status has prognostic and therapeutic implications for patients, with dMMR having a favourable impact on prognosis in early-stage CRC, but resulting in a worse prognosis in mCRC compared to proficient mismatch repair (pMMR) tumours.^1,4,5^ Immune checkpoint inhibitor (ICI) trials have shown a durable response in pretreated dMMR mCRC patients.^6–8^ dMMR tumours are highly sensitive to ICI due to the high mutational load, immune infiltrate and immune checkpoint signalling.^6^ Overman demonstrated a 1-year OS rate of 85% in dMMR mCRC patients, who were refractory to at least one systemic treatment line, upon treatment with nivolumab/ipilimumab.^8^ ICI have been approved by the Food and Drug Administration (FDA) in dMMR mCRC patients beyond first-line treatment. However, the European Medicines Agency (EMA) has not approved ICI based on the lack of a standard control arm without immunotherapy in the ICI trials. For a better interpretation of published immunotherapy results, survival data beyond first-line in dMMR mCRC patients who did not receive immunotherapy are needed.

Due to the low incidence of dMMR among mCRC patients, data on survival in dMMR mCRC patients receiving standard systemic treatments are scarce.^1,3,9–12^ Published data of survival beyond first-line treatment in a cohort of dMMR mCRC patients not receiving immunotherapy are highly needed. We report on the survival and factors affecting survival of a large cohort of dMMR mCRC patients.

## Methods

### Study population

We analysed dMMR mCRC patients in two populations: population-based patients registered in the Netherlands Cancer Registry (NCR) managed by the Netherlands Comprehensive Cancer Organisation (IKNL), and trial-based patients from three prospective Phase 3 first-line clinical trials (CAIRO,^13^ CAIRO2^14^ and CAIRO3^15^). For trial-based patients, patient inclusion criteria, informed consent and study protocols for the trials were published previously.^13–15^ The privacy rights for patients were maintained.

**Fig. 1:**
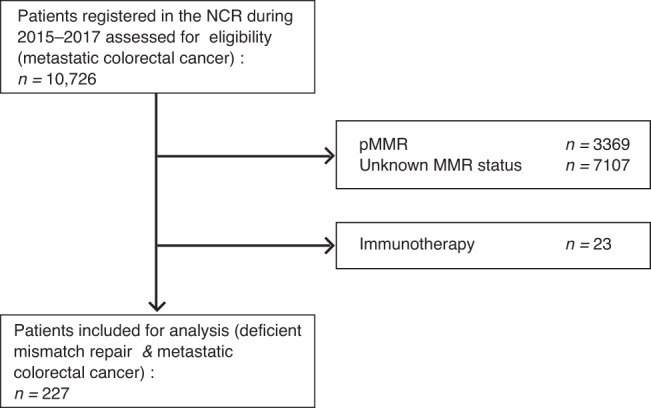
Flow diagram of Netherlands Cancer Registry (NCR) patients. All NCR patients with histologically proven metastatic colorectal cancer (mCRC) were assessed for eligibility. Patients were excluded if mismatch repair (MMR) status was unknown or proficient MMR (pMMR) and if patients received immunotherapy during treatment. The final population-based cohort consists of 227 patients with deficient mismatch repair (dMMR) mCRC.

Inclusion criteria for the current analysis were histologically proven mCRC with a dMMR tumour. Patients who received immunotherapy during the course of their disease were excluded. Clinical data of all newly diagnosed cancer patients in the Netherlands are registered in the NCR. dMMR mCRC patients with an incidence date between January 1, 2015 and December 31, 2017 were included, since mismatch repair status is registered in the NCR for patients with an incidence date only after 2015. Figure 1 is a flow diagram of the patients identified in the NCR cohort. dMMR status was known when determined in routine clinical practice during the period of data registration. NCR-derived patients include all synchronous and some metachronous mCRC patients with known dMMR status due to the data collection procedure of the NCR. For the same registration period, the overall survival and treatment status for pMMR mCRC NCR-derived patients was collected.

### Data collection

For population-based patients, pseudonymised clinical data on demographic characteristics, tumour characteristics and treatment information (type, response) were obtained from the NCR. Vital status for NCR patients was obtained using a yearly coupling with the municipal population registry to the cancer registry on February 1st, 2019. For NCR pMMR patients, clinical variables were obtained from the first registration period. For patients in the CAIRO trials, clinical data were available and follow-up information was updated up to October 2019. Data registration was completed for all dMMR patients, population-based and trial-based, by ensuring that no more than 30 days of clinical data was lacking prior to the vital status coupling. If data registration could not be completed, vital status was censored to the last known date of clinical data (*n* = 13).

Any *BRAF* mutation detected was included in the definition of *BRAF-*mutant status. Sidedness of the primary tumour was defined as right-sided (coecum-transverse colon), left-sided (splenic flexure-sigmoid) and rectosigmoid/rectal. Antitumour therapy was defined as systemic treatment (excluding adjuvant therapy) or local treatment (surgical resection of metastases, radiofrequency ablation (RFA), microwave ablation (MWA), HIPEC (Hyperthermic Intraperitoneal Chemotherapy) or PIPAC (Pressurized Intra Peritoneal Aerosol Chemotherapy)). Antitumour therapy was categorised as follows: no antitumour therapy, local treatment only, local and systemic treatment and systemic treatment only.

### Deficient mismatch repair

In the CAIRO trials dMMR status was determined according to the study protocol.^1,16,17^ In the NCR cohort, dMMR was determined according to Dutch guidelines in accredited laboratories, using immunohistochemistry and/or polymerase chain reaction.

### Study parameters

Overall survival was defined as the interval from diagnosis of metastatic disease until death of any cause or date of last follow-up if alive (OS). In patients receiving systemic treatment, OS was measured from each treatment line initiation, resulting in OS1 in patients receiving first-line systemic treatment, and similarly OS2 and OS3 in patients receiving second-line or third-line systemic treatment starting from second-line or third-line initiation, respectively.

Survival rates and patient characteristics were obtained from published ICI trials.^7,8^ In order to bring our results in perspective with ICI trials without a control arm, our results were reported alongside the most comparable ICI trials, which analysed survival from second-line.

### Statistical analysis

Baseline characteristics of the patients were analysed for the whole cohort and relevant differences between population-based and trial-based groups were described. Kaplan–Meier curves and 9-month and 12-month survival rate estimates were obtained for OS, OS1, OS2 and OS3. Subgroup analyses between population-based and trial-based patients were performed using the log-rank test.

Cox regression univariate and multivariable analysis was performed in patients receiving first-line treatment for OS. Ten preselected prognostic factors were selected: age at diagnosis of metastatic disease, gender, trial participation, *BRAF* mutation, primary tumour sidedness, metastatic sites location, stage at diagnosis, number of treatment lines given, primary tumour resection and metastasectomy.^3,10,18^ An unadjusted median overall survival (from diagnosis metastatic disease) for each level of the covariates was obtained by performing a log-rank test in patients receiving first-line systemic therapy. Multiple imputation by chained equations was used for covariates with missing data.^19^ From the complete dataset of variables, predictor variables with a correlation >0.20 with the missing variables and <15% missing values were selected to use alongside the ten covariates and Cox regression outcome variables in multiple imputation. Patients with missing data were compared to patients with complete-cases (Supplementary Table S1). Univariate hazard ratios for each covariate were obtained using Cox regression. A stratified Cox proportional hazards multivariable model was obtained using the preselected ten covariates for OS; stratified for the number of treatment lines received since this covariate violated the proportional hazards assumption. Regression analysis was performed on each imputed dataset and combined using Rubin’s rules.

All analyses were performed in R (version 3.5.1, “survival”, “survminer”, “mice” and “lattice” packages^20^).

## Results

### Patient characteristics

The cohort comprises 281 patients: 227 population-based (NCR) and 54 trial-based patients. The characteristics of the cohort are described in Table 1.

**Table 1:** Patient characteristics.

	dMMR cohort
	*n* = 281
Patient type	
Trial-based	54 (19.2)
Population-based	227 (80.8)
Age (%)	
≤55 years	59 (21.1)
56–65 years	57 (20.4)
66–75 years	103 (36.9)
>75 years	60 (21.5)
Female (%)	159 (56.6)
*BRAF* mutational status (%)	
Wildtype	68 (45.3)
Mutation	82 (54.7)
Unknown	131
*RAS* mutational status (%)	
Wildtype	104 (81.2)
Mutation	24 (18.8)
Unknown	153
Stage (%)	
I	2 (0.7)
II	28 (10.0)
III	88 (31.5)
IV	161 (57.7)
Sidedness (%)	
Right-sided	202 (72.9)
Left-sided	55 (19.9)
Rectosigmoid/rectum	20 (7.2)
Synchronous metastatic pattern (%)	194 (69.0)
Metastatic localisation (%)	
Liver-only	61 (21.7)
Extrahepatic	133 (47.3)
Peritoneal	87 (31.0)
Number of metastatic sites (%)	
1	166 (59.3)
2	71 (25.4)
3	33 (11.8)
≥4	10 (3.6)
Primary tumour resection (%)	219 (77.9)
Metastasectomy (%)	64 (22.8)
Local treatment metastases (%)	
RFA	6 (2.3)
MWA	1 (0.4)
HIPEC	27 (9.6)
Antitumour therapy (%)	
No treatment	72 (25.6)
Local treatment	36 (12.8)
Local and systemic treatment	38 (13.5)
Systemic treatment	135 (48.0)
Adjuvant chemotherapy (%)	47 (16.7)
Systemic therapy	
First-line treatment (%)	173 (61.6)
Second-line treatment (%)	72 (25.6)
Third-line treatment (%)	21 (7.5)
Fourth-line treatment (%)	3 (1.1)
WHO PS (at start first-line, % of first-line)	
Score 0	57 (52.2)
Score 1	45 (41.3)
Score ≥ 2	7 (6.4)
Unknown	64

Characteristics of patients at diagnosis of metastatic disease with treatment information during the course of disease. Trial-based patients were obtained from the CAIRO (*n* = 19), CAIRO2 (*n* = 31) and CAIRO3 (*n* = 4) phase III randomised controlled trials. Sidedness of the primary tumour was defined as right-sided (coecum-transverse colon), left-sided (splenic flexure-sigmoid) and rectosigmoid/rectal. Local treatment was defined as metastasectomy or local metastatic treatment (RFA, MWA or HIPEC/PIPAC) and systemic treatment as all systemic therapy given for metastatic disease (excluding adjuvant therapy). Missing values are not shown if missing frequency was less than 5%.

*WHO PS*: World Health Organisation Performance Score, percentages relative to amount of people receiving first-line treatment.

Of the 281 patients, 57% were female, 73% had a right-sided tumour. Age had a bimodal distribution around 50 and 70 years. Of patients with a known *BRAF* mutation status, 55% (*n* = 82/150) had a *BRAF* mutation. A primary tumour resection and metastasectomy was performed in 78% and 23% of patients, respectively. Of patients with a known WHO performance score at start of first-line treatment, 93% (*n* = 102/109) had a WHO performance score of 0–1, with an unknown performance score in 64 patients.

In our cohort, 26% (*n* = 72) of patients received no antitumour treatment, 13% (*n* = *36*) received local treatment, 14% (*n* = 38) received local and systemic treatment and 48% (*n* = 135) received only systemic treatment (Table 1). Sixty-two percent of patients received first-line, 26% second-line and 8% third-line systemic non-immunotherapy. The type of treatment regimens and agents used per treatment line are described in Supplementary Table S2.

Comparing patient characteristics between population-based (2015–2018) and trial-based patients (2002–2011): population-based patients were older at diagnosis of metastatic disease (age above 75 years in 24% versus 10%), had less primary tumour resections (74% versus 96%) and more often resection of metastases (26% versus 9%), as shown in Supplementary Table S3. All trial-based patients were recruited from intervention trials with first-line systemic therapy, which is reflected in the proportion of patients receiving systemic therapy (100% trial-based versus 52% population-based).

### Follow-up

For the population-based and trial-based cohort, the median follow-up period from diagnosis of metastatic disease was 8.3 months (interquartile range [IQR] 3.1–17.6) and 16.8 months (9.5–22.8), respectively. At the end of the follow-up period, 70.1% of patients were deceased, which was an indication that follow-up was adequate. Follow-up was completed for 264 patients (94%).

### OS during treatment

**Fig. 2:**
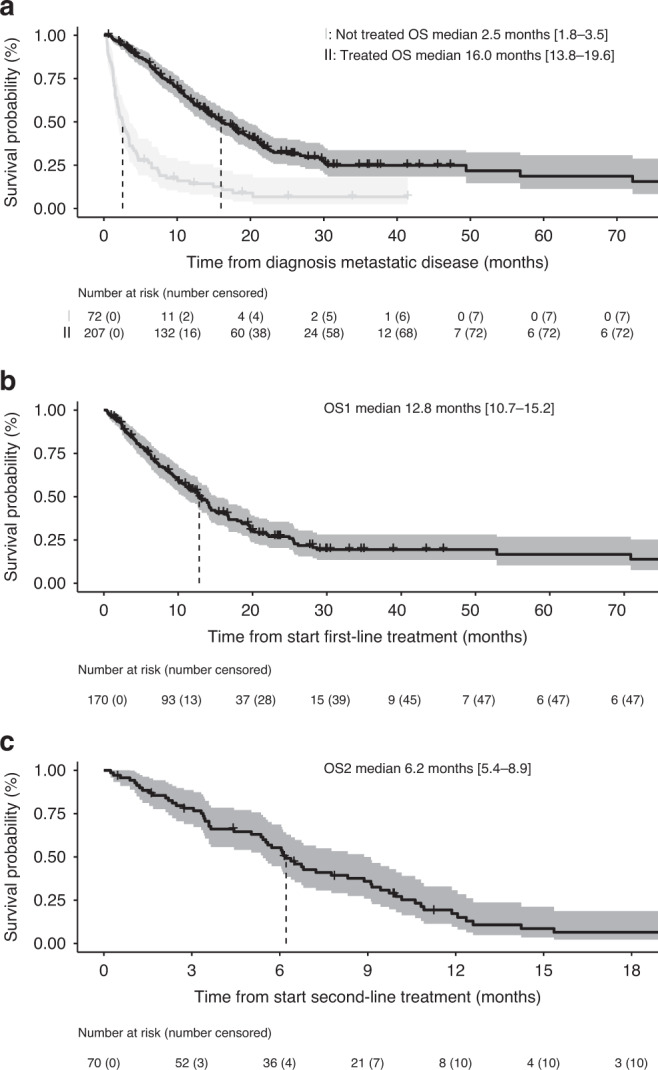
OS, OS1 and OS2 in mCRC patients with dMMR. Predicted overall survival (OS) using Kaplan–Meier curves with confidence intervals, examined for the cohort as a whole and in patients who received systemic treatment. **a** OS from metastatic disease in the unselected dMMR cohort (*n* = *279*), stratified by having received antitumour therapy (*n* = 207) versus no antitumour therapy (*n* = 72), **b** Overall survival from first-line systemic therapy initiation (OS1) in first-line patients (*n* = 170), **c** Overall survival from second-line systemic therapy initiation (OS2) in second-line patients (*n* = 70). The risk tables display the number of patients at risk and the number of censored patients. Median survival is indicated with a vertical dashed line in each plot and is indicated with 95% confidence interval lower and upper ranges in the legend.

The Kaplan–Meier survival curves from diagnosis of metastatic disease and from start of each therapy line are demonstrated in Fig. 2. We describe survival for patients who received antitumour therapy (systemic treatment, excluding adjuvant therapy, surgical resection of metastases and/or local treatment of metastases (including HIPEC or PIPAC)) versus patients who did not receive antitumour therapy. For patients who received antitumour therapy, median OS was 16.0 months (95% Confidence Interval [C.I.] 13.8–19.6; *n* = 207), whereas OS was 2.5 months (95% C.I. 1.8–3.5; *n* = 72) in patients without antitumour therapy. Furthermore, examining survival per type of treatment received, median OS was longer in patients receiving local and systemic therapy (median OS 29.9 months, 95% C.I. 17.9-not reached; *n* = *38*) compared to patients receiving only systemic therapy (13.9 months, 95% C.I. 11.4–16.5; *n* = *133*), as shown in Supplementary Table S4. Compared to the other treatment categories, patients receiving local and systemic treatment more often had primary rectal tumours (19% compared to <7% in other categories), *BRAF* wildtype tumours (65% compared to <49% in other categories) and were younger (≤55 years in 42% versus <23% in other categories), as shown in Supplementary Table S5. Median OS increased in patients who received more systemic treatment lines (Supplementary Table S4).

In order to examine the OS from initiation of each treatment line, comprising only patients receiving the given treatment line, we examined the OS1, OS2 and OS3. The median overall survival from first-line systemic therapy initiation (OS1) was 12.8 months (95% C.I. 10.7–15.2; *n* = 170), from second-line systemic therapy initiation (OS2) 6.2 months (95% C.I. 5.4–8.9; *n* = 70) and from third-line systemic therapy initiation (OS3) 3.6 months (95% C.I. 2.7–not reached; *n* = 19).

From first-line systemic therapy initiation (OS1), estimated 9-month and 12-month survival rates were 63.6% (95% C.I. 56.6–71.5) and 53.8% (95% C.I. 46.5–62.1). Similarly, from second-line initiation (OS2), 9-month and 12-month survival rates were 35.9% (95% C.I. 25.9–49.9) and 17.2% (95% C.I. 9.7–30.7). In patients who received second-line systemic treatment with a known WHO performance score ≤1, similar to the inclusion criteria of the CheckMate 142 trials (*n* = 24),^7,8^ predicted 9-month and 12-month survival rates from start of second-line systemic therapy were 43.6% (95% C.I. 27.4–69.4) and 17.4% (95% C.I. 7.2–42.4), respectively. Supplementary Table S6 demonstrates the characteristics of our second-line patients alongside the nivolumab and nivolumab/ipilimumab CheckMate 142 trial cohorts.

We compared the survival between population-based and trial-based dMMR patients, examining differences in OS among treated patients, OS1 and OS2. There was no significant difference in log-rank comparison of population-based versus trial-based patients in OS, OS1 or OS2. The median OS was 16.0 months (95%C.I. 13.0–22.1; *n* = 155) and 16.8 months (95% C.I. 13.5–21.0; *n* = 52; *p* = 0.27) in population-based and trial-based patients, respectively. Similarly, the median OS1 was 12.6 months (95% C.I. 10.1–15.0; *n* = 118 population-based) and 13.5 months (95% C.I. 9.1–19.6; *n* = 52 trial-based; *p* = 0.59). The median OS2 was 6.1 months (95% C.I. 4.4–8.9) in 43 population-based patients and 6.7 months (95% C.I. 5.0–10.2; *p* = 0.58) in 27 trial-based patients.

For population-based patients, we compared the median OS between patients with dMMR tumours versus pMMR tumours. For trial-based patients this was previously published, showing that the median OS was shorter in dMMR versus pMMR trial-based patients.^1,17^ For population-based patients, the median OS was significantly shorter in patients with dMMR tumours upon receiving antitumour therapy than pMMR tumours (Table 2). The median OS was 7.6 months shorter, with a median OS of 16.0 months (95% C.I. 13.0–22.1; *n* = 155) for dMMR tumours compared to 23.6 months (95%C.I. 22.6–24.6; *n* = 2746; *p* < 0.005) for pMMR tumours in patients receiving treatment. In untreated patients, median OS was 2.5 months (95% C.I. 1.8–3.5; *n* = 72) for dMMR tumours versus 3.9 months (95% C.I. 3.4–4.8; *n* = 610*; p* = 0.005) for pMMR tumours.

**Table 2:** Overall survival in population-based metastatic colorectal cancer patients.

Antitumour therapy	Mismatch repair status	Median OS (months)	*p* value
Treated	dMMR (*n* = 155)	16.0 [13.0–22.1]	<0.005
	pMMR (*n* = 2746)	23.6 [22.6–24.6]	
Untreated	dMMR (*n* = 72)	2.5 [1.8–3.5]	0.005
	pMMR (*n* = 610)	3.9 [3.4–4.8]	

Population-based patients with metastatic colorectal cancer and known mismatch repair status, registered between 2015 and 2017 in the Netherlands Cancer Registry. OS was measured from diagnosis of metastatic disease until vital status coupling and is indicated with the 95% confidence interval. Log-rank test was performed for untreated patients (dMMR versus pMMR) and for treated patients (dMMR versus pMMR). Antitumour therapy was defined as systemic treatment (with exception of adjuvant chemotherapy), metastasectomy or local metastatic treatment (RFA, MWA or HIPEC/PIPAC).

*dMMR* deficient mismatch repair, *HIPEC* hyperthermic intraperitoneal chemotherapy, *MWA* microwave ablation, *OS* overall survival, *PIPAC* pressurised intraperitoneal aerosol chemotherapy, *pMMR* proficient mismatch repair, *RFA* radiofrequency ablation.

### Prognostic variables associated with overall survival in patients receiving first-line systemic treatment

In univariate analysis, metastasectomy and sidedness were significantly associated with OS from diagnosis of metastatic disease in patients receiving first-line systemic treatment (Table 3). *BRAF* mutational status had a higher risk for shorter OS, albeit nonsignificant, in univariate analysis (unadjusted hazard ratio [HR] 1.51 (95% C.I. 1.00–2.28); *p* = 0.052) as shown in Table 3.

**Table 3:** Univariate and multivariable cox regression models for overall survival in first-line treated patients.

			Univariate			Multivariable	
Variable	Level	*n*	OS in months [95% C.I.]^KM^	Hazard ratio (95% C.I.)^CPH^	*p* value	Hazard ratio (95% C.I.)^CPH^	*p* value
**First-line patients**		**171**	**15.3 [13.1–18.3]**				
Age	≤65 years	83	18.0 [13.9***–***22.4]				
	>65 years	88	13.8 [10.9***–***17.6]	1.409 (0.979–2.027)	0.065	1.075 (0.709–1.628)	0.734
Gender	Male	86	15.9 [12.3***–***21.0]				
	Female	85	14.6 [11.8***–***18.0]	1.238 (0.861–1.782)	0.249	0.965 (0.655–1.421)	0.857
* BRAF*	Wildtype	63	19.6 [14.6***–***22.7]				
	Mutation	59	11.5 [8.7***–***17.9]	1.509 (0.998–2.282)	0.052	1.610 (0.936–2.771)	0.086
Metastatic sites	Hepatic	39	15.9 [12.2***–***23.1]				
	Extrahepatic	85	14.6 [11.1***–***21.0]	1.012 (0.651–1.574)	0.957	0.859 (0.527–1.399)	0.540
	Peritoneal	47	15.2 [12.3***–***21.6]	1.017 (0.601–1.721)	0.950	0.814 (0.447–1.484)	0.502
Metastasectomy	No	136	14.1 [11.5***–***17.2]				
	Yes	35	29.5 [17.9***–***NR]	0.454 (0.271–0.761)	**0.003**^a^	0.486 (0.262–0.903)	**0.022**^a^
Primary tumour resection	No	45	14.1 [11.1***–***17.6]				
	Yes	126	16.5 [13.5***–***21.0]	0.664 (0.437–1.007)	0.054	0.628 (0.365–1.083)	0.094
Primary tumour location	Left-sided/rectosigmoid/rectum	53	21.6 [16.5***–***72.1]				
	Right-sided	114	13.8 [10.9***–***17.2]	1.663 (1.112–2.486)	**0.013**^a^	1.705 (1.042–2.790)	**0.034**^a^
Stage at diagnosis	Stage I/II	16	14.6 [10.3***–***NR]				
	Stage III	39	14.6 [7.1***–***20.8]	1.293 (0.655–2.550)	0.459	0.944 (0.447–1.996)	0.881
	Stage IV	113	16.0 [13.1***–***19.8]	0.980 (0.532–1.805)	0.948	0.723 (0.351–1.490)	0.380
Cohort	Population-based	119	15.2 [12.3***–***18.3]				
	Trial-based	52	16.8 [13.5***–***21.0]	1.096 (0.752–1.598)	0.633	0.728 (0.434–1.219)	0.227
Treatment lines received	<2	100	13.8 [9.9***–***19.6]				
	≥2	71	16.0 [14.1***–***21.0]	1.121 (0.780–1.610)	0.538		

Multivariable results were calculated stratified per number of treatment lines received (which had violated the assumption of proportional hazards) using the imputed dataset. An unadjusted median overall survival from diagnosis metastatic disease was obtained from a Kaplan–Meier curve stratified for the given variable using the original dataset. The counts (n) reflect the counts of the patients used in the survival analysis (non-imputed dataset), which may differ from the total cohort due to missing data in the variable or outcome.

Bold numbers represent statistically significant hazard ratios.

*95% C.I* 95% confidence interval, *CPH* Cox Proportionate Hazards Model, *HR* hazard ratio, *KM* Kaplan–Meier, *NR* not reached, *OS* overall survival.

^a^Indicates statistically significant hazard ratio’s (*p* value < 0.05).

The final multivariable model was a stratified Cox regression model for the number of treatment lines received (≤2 and >2) since the variable violated the proportional hazards assumption. In the stratified Cox regression model for number of treatment lines received, metastasectomy is significantly associated with a longer survival (hazard ratio [HR] 0.49 (95% C.I. 0.26–0.90); *p* < 0.05) and right-sided tumour location is significantly associated with a shorter survival (HR1.71 (95% C.I. 1.04–2.79); *p* < 0.05) as shown in Table 3. In patients receiving first-line systemic treatment, the unadjusted median OS was 29.5 months (95% C.I. 17.9– not reached; *n* = 35) and 14.1 months (95% C.I. 11.5–17.2; *n* = 136) for patients with and without a metastasectomy, respectively (Table 3). Similarly, the unadjusted median OS was 13.8 months (95% C.I. 10.9–17.2; *n* = 114) versus 21.6 months (95% C.I. 16.5–72.1; *n* = 53) in patients receiving first-line systemic treatment with a right-sided versus left-sided primary tumour location. *BRAF* mutational status had a higher risk for shorter OS, albeit nonsignificant, in multivariable analysis (HR1.61 (95% C.I. 0.94–2.77); *p* = 0.086), with an unadjusted median OS of 11.5 months (95% C.I. 8.7–17.9; *n* = 59) in patients receiving first-line systemic treatment with a *BRAF*-mutant tumour versus 19.6 months (95% C.I. 14.6–22.7; *n* = 63) in patients with a *BRAF*-wildtype tumour. The other covariates were not significantly associated with survival in the final multivariable model.

## Discussion

We present survival data of a large, comprehensive cohort of dMMR mCRC patients, not treated with immunotherapy. Our cohort offers a unique insight into the survival of dMMR mCRC patients while receiving systemic non-immunotherapy in first-, second- and third-line treatment. The OS in our dMMR mCRC cohort for all patients and patients receiving first-line treatment is comparable to previously reported survival data in dMMR mCRC patients without immunotherapy, including two population-based dMMR mCRC cohorts with a similar percentage of patients receiving systemic therapy.^5,9,10,12,21^ However, the OS in our dMMR mCRC patients is shorter than the median OS in three other publications, which ranged from 26–39 months.^3,11,22^ The difference may be due to the patient characteristics in the cohorts, with cohorts including patients receiving immunotherapy,^22^ a high proportion (44–63%) of Lynch syndrome (*BRAF* wildtype) patients,^3,22^ and a high proportion (57%) of patients who underwent a metastasectomy.^11^ The median OS from initiation of second-line treatment (6.2 months) in our cohort is drastically shorter than the recently reported median OS in second-line patients (21.6 months) in a cohort of dMMR mCRC patients receiving systemic non-immunotherapy and immunotherapy.^22^ The difference may be due to our cohort comprising only patients receiving systemic non-immunotherapy, while the Tougeron et al. dMMR mCRC patient cohort included patients receiving immunotherapy and a high proportion (44%) of Lynch syndrome patients.^22^

In our population-based patients, the median OS during treatment was significantly shorter in dMMR compared to pMMR mCRC patients. This supports previous studies reporting a worse survival in mCRC patients with dMMR^1,5,12^ and is in contrast to studies showing a nonsignificant, null or opposite effect on survival.^9,11,21,23^ The conflicting results may lie in heterogeneity of the population cohort being studied, with studies finding a null or opposite effect on survival having included patients with a low percentage of *BRAF* mutations,^9^ only metachronous disease^23^ or younger patients.^11^ Our population reflects a clinically relevant cross-sectional population of Dutch patients who received MMR testing, indicating that patients with mCRC and known dMMR have a worse prognosis compared to pMMR patients.

In patients treated with at least one line of systemic treatment, we observed a significant association between metastasectomy with better survival and right-sided primary tumour location at diagnosis (‘sidedness’) with worse survival. In unselected mCRC patients and dMMR mCRC patients, metastasectomy is a known prognostic factor for OS.^3,10,24^ Sidedness is an important prognostic factor in mCRC patients. However, sidedness has not yet been shown to be associated with OS in patients with dMMR tumours.^3,24^ Our results indicate that in dMMR mCRC patients, right-sidedness is associated with worse survival. Patients receiving first-line systemic treatment with a *BRAF* mutation had a higher risk for shorter survival in multivariable analysis, although nonsignificant, which is reflected with an 8-month difference in the unadjusted median OS in patients with a *BRAF* mutant versus *BRAF*-wildtype tumour. Studies have demonstrated that *BRAF* mutational status was prognostic within dMMR mCRC patients;^10,23^ however, this is not consistently shown.^5,22^ Although we did not identify a significant association for *BRAF* mutation with survival in patients receiving first-line systemic treatment, our results suggest that patients with a *BRAF* mutation do have a higher risk for shorter survival.

In addition to the prognostic factors which we examined in dMMR mCRC patients receiving first-line systemic treatment, other factors may contribute to the worse prognosis seen in dMMR mCRC patients. Population-based dMMR mCRC patients have a lower response rate to first-line systemic non-immunotherapy compared to pMMR mCRC patients (5% versus 44%, respectively) and are also less likely to receive systemic therapy compared to pMMR mCRC patients (47% versus 73%, *p* < 0.001).^5,21^ Thus, the worse prognosis in dMMR mCRC patients is likely driven by several factors, potentially including primary tumour sidedness, *BRAF* mutational status, the response rate to systemic therapy, the ability to receive a metastatic resection, and other less well known factors, such as the PD-L1 gene expression level, reflecting immune evasion.^25^ Additionally, although dMMR mCRC patients are often analysed as one entity, different subgroups with different prognosis should be identified to compare survival results between studies including Lynch syndrome (often *BRAF*-wildtype), sporadic *BRAF* mutated dMMR tumours and sporadic *BRAF-*wildtype dMMR tumours.^3,9,10^ Although *BRAF* status and MMR status was known, due to unavailable data regarding MLH1 methylation and Lynch syndrome status we were unable to distinguish between sporadic versus Lynch origin.

We are aware of several limitations. Although we were able to include a broad range of relevant variables, we cannot exclude confounding from unmeasured variables. Secondly, our retrospective study design may have resulted in a selection of the population, since MMR status was not determined in all patients in daily practice. Lastly, comparison of our data with other studies may be confounded by differences in patient characteristics.

Our study is unique in providing survival data on a large cohort of population-based and trial-based dMMR mCRC patients in the pre-immunotherapy era. Our survival data of dMMR mCRC patients beyond first-line treatment may be compared with for instance the CheckMate 142 trial results, which examined nivolumab and nivolumab/ipilimumab treatment in dMMR mCRC patients beyond first-line treatment.^7,8^ The 9-month and 12-month survival rates of patients receiving second-line treatment in our cohort of 35.9% and 17.2%, respectively, are lower than the published 9-month survival rate in the nivolumab/ipilimumab arm of 87%, the 12-month survival rates in the nivolumab arm of 73% and the nivolumab/ipilimumab arm of 85%.^7,8^ The cohorts are comparable in key patient and tumour characteristics, although the immunotherapy cohorts were more heavily pretreated (40–54% receiving ≥3 treatment lines in the CheckMate 142 trials compared to 29% in our cohort) and patients more often having *BRAF*-wildtype status. Both characteristics may reflect a patient selection in the CheckMate 142 trials with a less aggressive clinical course compared to our cohort. Still, even the heavily treated patients in our cohort (who had received ≥3 treatment lines) had a median OS of only 18 months. However, as we had no access to individual patient data of the other cohorts, a direct comparison between the cohorts was not possible. A comparison with the Phase 2 pembrolizumab trial was not possible due to the low number of patients in our cohort who received third-line treatment.^6^ The CheckMate 142 results for patients receiving nivolumab/ipilimumab in first-line setting suggest a benefit of immunotherapy compared to our systemic non-immunotherapy first-line cohort, with a median 12-month OS rate of 83% versus 54%, respectively.^26^ This is supported by the Keynote-177 Phase 3 randomised controlled trial results, which show that dMMR mCRC patients have a PFS benefit when receiving first-line pembrolizumab versus first-line systemic therapy (mFOLFOX6 or FOLFIRI combined with bevacizumab or cetuximab), with a median PFS of 16.5 months versus 8.2 months, respectively (HR0.60, 95% C.I. 0.45–0.80, *p* = 0.0002).^27^

In conclusion, we present survival data on a large, comprehensive cohort of dMMR mCRC patients, treated with or without systemic non-immunotherapy. Currently, available data from immunotherapy trials lack a control arm with standard systemic treatment. We demonstrate a poor prognostic value for dMMR in mCRC patients. Given the poor outcome in our dataset compared to the results of immunotherapy in dMMR mCRC patients, our data strongly support a survival benefit of immunotherapy in dMMR mCRC patients.

## Supplementary information

Supplementary files

## Data Availability

The datasets generated during and analyzed during the current study are not publicly available due to the regulations of the Netherlands Cancer Registry but are available from the corresponding author or Netherlands Cancer Registry on reasonable request.
