# Supplementary material for: Survival of patients with deficient mismatch repair metastatic colorectal cancer in the pre-immunotherapy era
Source: Br J Cancer. 2020 Oct 13;124(2):399–406. doi: 10.1038/s41416-020-01076-0 (PMC7852682; doi:10.1038/s41416-020-01076-0)
Supplement: Supplementary file 1 — Supplementary files [file 41416_2020_1076_MOESM1_ESM.pdf]

SUPPLEMENTARY TABLES

Files in this data supplement:

Supplementary Table S1. Characteristics of incomplete and complete cases in first-line treated patients

Supplementary Table S2. Systemic therapy regimen for patients

Supplementary Table S3. Characteristics of population- and trial-based patients

Supplementary Table S4. Overall survival per treatment group and treatment lines received

Supplementary Table S5. Characteristics of patients per type of anti-tumour treatment received

Supplementary Table S6. Comparison of non-immunotherapy versus immunotherapy cohorts

Supplementary Table S1. Characteristics of incomplete and complete cases in first-line treated patients

	Original dataset<br>*n=171*	Complete cases<br>*n=122*	Incomplete cases<br>*n=49*
**Age >65 years (%)**	88 (51·5)	61 (50·0)	27 (55·1)
**Trial-based (%)**	52 (30·4)	50 (41·0)	2 (4·1)
**Female (%)**	85 (49·7)	59 (48·4)	26 (53·1)
***BRAF* (%)**			
***BRAF* mutation**	59 (34·5)	59 (48·4)	
**Unknown**	49 (28·7)		49 (100·0)
**Stage (%)**			
**I/II**	17 (9·9)	12 (9·8)	5 (10·2)
**III**	39 (22·8)	28 (23·0)	11 (22·4)
**IV**	113 (66·1)	81 (66·4)	32 (65·3)
**Unknown**	2 (1·2)	1 (0·8)	1 (2·0)
**Sidedness (%)**			
**Left-sided**	53 (31·0)	34 (27·9)	19 (38·8)
**Right-sided**	114 (66·7)	85 (69·7)	29 (59·2)
**Unknown**	4 (2·3)	3 (2·5)	1 (2·0)
**Metastatic localization (%)**			
**Extra-hepatic**	85 (49·7)	64 (52·5)	21 (42·9)
**Liver-only**	39 (22·8)	31 (25·4)	8 (16·3)
**Peritoneal**	47 (27·5)	27 (22·1)	20 (40·8)
**Primary tumor resection (%)**	126 (73·7)	90 (73·8)	36 (73·5)
**Metastasectomy (%)**	35 (20·5)	21 (17·2)	14 (28·6)
**Adjuvant chemotherapy (%)**	21 (12·3)	15 (12·3)	6 (12·2)
**2 or more treatment lines (%)**	71 (41·5)	60 (49·2)	11 (22·4)

Characteristics of first-line patients in different cohorts are reported: A) all first-line patients with known survival status from the original dataset (*n=171*), B) subgroup of patients with complete *BRAF* cases (*n=122*), and C) subgroup of patients with incomplete *BRAF* cases (*n=49*).

Supplementary Table S2. Systemic therapy regimen for patients

	**Cohort** *n=281*
**First-line regimen** (% of cohort)	173 (61·6)
Monotherapy (% of first-line)	35 (20·2)
Doublet	132 (76·3)
Triplet	5 (2·9)
Chemotherapeutic agents (% of first-line)	
Capecitabine	34 (19·7)
CAPOX/FOLFOX	113 (65·3)
Irinotecan/CAPIRI/FOLFIRI	20 (11·6)
FOLFOXIRI	5 (2·9)
Other	1 (0.6)
Targeted agents (% of first-line)	
Anti-VEGF	89 (51·4)
Anti-EGFR	3 (1·7)
Combined anti-VEGF + EGFR	13 (7·5)
**Second-line regimen** (% of cohort)	72 (25·6)
Monotherapy (% of second-line)	50 (69·4)
Doublet	21 (29·2)
Chemotherapeutic agents (% of second-line)	
Capecitabine/UFT	2 (2·8)
CAPOX/FOLFOX	5 (6·9)
Irinotecan /CAPIRI/FOLFIRI	55 (76·4)
FOLFOXIRI	1 (1·4)
Other	1 (1·4)
Targeted agents (% of second-line)	
Anti-VEGF	6 (8·3)
Anti-EGFR	11 (15·3)
**Third-line regimen** (% of cohort)	21 (7·5)
Monotherapy (% of third-line)	14 (66·7)
Doublet	5 (23·8)
Chemotherapeutic agents (% of third-line)	
Capecitabine/UFT	1 (4·8)
Oxaliplatin/CAPOX/FOLFOX	6 (28·6)
Irinotecan/CAPIRI/FOLFIRI	1 (4·8)
Other	3 (14·3)
Targeted agents (% of third-line)	
Anti-VEGF	1 (4·8)
Anti-EGFR	8 (38·1)

Characteristics of patient treatment regimens for the whole follow-up period, reported for all patients receiving systemic therapy for palliative disease, including trial and population-based patients. Abbreviations: CAPIRI (capecitabine and irinotecan), CAPOX (capecitabine and oxaliplatin), EGFR (epidermal growth factor receptor), FOLFIRI (5-fluoro-uracil and irinotecan), FOLFOX (5-fluoro-uracil and oxaliplatin), FOLFOXIRI (5-fluoro-uracil, oxaliplatin and irinotecan), UFT (tegafur/uracil), and VEGF (vascular endothelial growth factor).

Supplementary Table S3. Characteristics of population- and trial-based patients

		**Population-based** *n=227*	**Trial-based** *n=54*	***p*-value**
**Age (%)**	≤ 55 years	47 (20·7)	12 (23·1)	*0·120*
	56-65 years	43 (18·9)	14 (26·9)	
	66-75 years	82 (36·1)	21 (40·4)	
	> 75 years	55 (24·2)	5 (9·6)	
**Female (%)**		134 (59·0)	25 (46·3)	*0.123*
***BRAF* mutational status (%)**	Wild-type	42 (42·0)	26 (52·0)	*0·324*
	Mutation	58 (58·0)	24 (48·0)	
	Unknown	127	4	
***RAS* mutational status (%)**	Wild-type	61 (78·2)	43 (86·0)	*0·384*
	Mutation	17 (21·8)	7 (14·0)	
	Unknown	149	4	
**Stage (%)**	I	1 (0·4)	1 (1·9)	*0·141*
	II	19 (8·4)	9 (17·0)	
	III	73 (32·3)	15 (28·3)	
	IV	133 (58·8)	28 (52·8)	
**Sidedness (%)**	Right-sided	160 (71·4)	42 (79·2)	*0·565*
	Left-sided	47 (21·0)	8 (15·1)	
	Rectosigmoid/Rectum	17 (7·6)	3 (5·7)	
**Synchronous metastatic pattern (%)**	Synchronous	158 (69·6)	36 (66·7)	*0·798*
**Metastatic localization (%)**	Liver-only	46 (20·3)	15 (27·8)	***<0·001***
	Extra-hepatic	97 (42·7)	36 (66·7)	
	Peritoneal	84 (37·0)	3 (5·6)	
**Number of metastatic sites (%)**	1	142 (62·6)	24 (45·3)	*0·105*
	2	53 (23·3)	18 (34·0)	
	3	24 (10·6)	9 (17·0)	
	≥4	8 (3·5)	2 (3·8)	
**Primary tumor resection (%)**		167 (73·6)	52 (96·3)	***<0·001***
**Metastasectomy (%)**		59 (26·0)	5 (9·3)	***0·014***
**Local treatment metastases (%)**	RFA	5 (2·2)	1 (1·9)	***0·012***
	MWA	1 (0·4)	0 (0·0)	
	HIPEC	27 (11·9)	0 (0·0)	
**Anti-tumour therapy (%)**	No treatment	72 (31·7)	0 (0·0)	***<0·001***
	Local treatment	36 (15·9)	0 (0·0)	
	Local and systemic treatment	33 (14·5)	5 (9·3)	
	Systemic treatment	86 (37·9)	49 (90·7)	
**Adjuvant chemotherapy (%)**		39 (17·2)	8 (14·8)	*0·829*
**Systemic therapy**	1st-line (%)	119 (52·4)	54 (100·0)	***<0·001***
	2nd-line (%)	43 (18·9)	29 (53·7)	***<0·001***
	3rd-line (%)	12 (5·3)	9 (16·7)	***0·010***
	4th-line (%)	3 (1·3)	0 (0·0)	*1·000*

Characteristics of patients at diagnosis of metastatic disease with treatment information during the course of disease. Trial-based patients were obtained from the CAIRO (n=19), CAIRO2 (n=31) and CAIRO3 (n=4) phase III randomized controlled trials. ** Indicates statistically significant hazard ratio's (p-value <0·05). Abbreviations: Sidedness of the primary tumour was defined as right-sided (coecum-transverse colon), left-sided (splenic flexure-sigmoid) and rectosigmoid/rectal. Local treatment was defined as metastasectomy or local metastatic treatment (RFA, MWA or HIPEC/PIPAC) and systemic therapy as all systemic treatment given for metastatic disease (excluding adjuvant therapy). Missing values are not shown if missing frequency was less than 5%. *p*-values are shown for comparison between population-based and trial-based patients (default test is the Chi-squared test, except for variables with <5 events per cell, where a Fisher's Exact test was performed).

Supplementary Table S4. Overall survival per treatment group and treatment lines received

		**Overall Survival** (median months with 95% C.I.)
All patients	*n = 279*	11·8 [10·1 - 14·6]
No tumour-directed therapy	*n = 72*	2·5 [1·8 - 3·5]
Tumour-directed therapy	*n = 207*	16·0 [13·8 - 19·6]
Received local treatment	*n = 36*	NR [11·8 - NR]
Received local and systemic treatment	*n = 38*	29·9 [17·9 - NR]
Received systemic treatment	*n = 133*	13·9 [11·4 - 16·5]
Received first-line systemic therapy	*n = 171*	15·3 [13·1 - 18·3]
Received second-line systemic therapy	*n = 70*	16·0 [14·1 - 21·0]
Received third-line systemic therapy	*n = 21*	18·0 [13·1 - 29·9]

Overall survival from diagnosis of metastatic disease until death or last follow-up if alive for patients with known survival data (*n=2* missing in cohort). Tumour-directed treatment in metastatic setting was defined as systemic treatment, metastasectomy or local metastatic treatment (radio-frequency ablation (RFA), microwave ablation (MWA), hyperthermic intraperitoneal chemotherapy (HIPEC) or pressurized intraperitoneal aerosol chemotherapy (PIPAC)). Local treatment was defined as metastasectomy or local metastatic treatment (RFA, MWA or HIPEC/PIPAC). Systemic therapy is defined as all systemic treatment given for metastatic disease, excluding adjuvant systemic therapy. Abbreviations: C.I. (Confidence Interval), NR (not reached).

Supplementary Table S5. Characteristics of patients per type of anti-tumour treatment received

		**No treatment** *n=72*	**Local treatment** *n=36*	**Local & systemic treatment** *n=38*	**Systemic treatment** *n=135*
**Age (%)**	≤ 55 years	7 (9·7)	6 (16·7)	16 (42·1)	30 (22·6)
	56-65 years	11 (15·3)	9 (25·0)	9 (23·7)	28 (21·1)
	66-75 years	28 (38·9)	14 (38·9)	11 (28·9)	50 (37·6)
	> 75 years	26 (36·1)	7 (19·4)	2 (5·3)	25 (18·8)
**Female (%)**		49 (68·1)	24 (66·7)	19 (50·0)	67 (49·6)
***BRAF* mutational status (%)**	Wild-type	4 (20·0)	1 (12·5)	15 (65·2)	48 (48·5)
	Mutation	16 (80·0)	7 (87·5)	8 (34·8)	51 (51·5)
	Unknown	52	28	15	36
***RAS* mutational status (%)**	Wild-type	8 (80·0)	2 (66·7)	14 (70·0)	80 (84·2)
	Mutation	2 (20·0)	1 (33·3)	6 (30·0)	15 (15·8)
	Unknown	62	33	18	40
**Stage (%)**	I	1 (1·4)	0 (0·0)	0 (0·0)	1 (0·7)
	II	7 (9·7)	5 (13·9)	2 (5·6)	14 (10·4)
	III	34 (47·2)	15 (41·7)	6 (16·7)	33 (24·4)
	IV	30 (41·7)	16 (44·4)	28 (77·8)	87 (64·4)
**Sidedness (%)**	Right-sided	57 (79·2)	29 (80·6)	21 (56·8)	95 (72·0)
	Left-sided	11 (15·3)	7 (19·4)	9 (24·3)	28 (21·2)
	Rectosigmoid/ Rectum	4 (5·6)	0 (0·0)	7 (18·9)	9 (6·8)
**Synchronous metastatic pattern (%)**		46 (63·9)	17 (47·2)	31 (81·6)	100 (74·1)
**Metastatic localization (%)**	Liver-only	13 (18·1)	9 (25·0)	13 (34·2)	26 (19·3)
	Extra-hepatic	35 (48·6)	12 (33·3)	12 (31·6)	74 (54·8)
	Peritoneal	24 (33·3)	15 (41·7)	13 (34·2)	35 (25·9)
**Number of metastatic sites (%)**	1	44 (61·1)	30 (83·3)	29 (76·3)	63 (47·0)
	2	18 (25·0)	4 (11·1)	5 (13·2)	44 (32·8)
	3	7 (9·7)	1 (2·8)	4 (10·5)	21 (15·7)
	≥4	3 (4·2)	1 (2·8)	0 (0·0)	6 (4·5)

Characteristics of patients at diagnosis of metastatic disease with treatment information during the course of disease. Abbreviations: World Health Organisation Performance Score (WHO PS, percentages relative to amount of people receiving first-line treatment). Sidedness of the primary tumour was defined as right-sided (coecum-transverse colon), left-sided (splenic flexure-sigmoid) and rectosigmoid/rectal. Local treatment was defined as metastasectomy or local metastatic treatment (radio-frequency ablation (RFA), microwave ablation (MWA), hyperthermic intraperitoneal chemotherapy (HIPEC) or pressurized intraperitoneal aerosol chemotherapy (PIPAC)). Missing values are not shown if missing frequency was less than 5%.

Supplementary Table S6. Comparison of non-immunotherapy versus immunotherapy cohorts

	**Current cohort: Second-line patients** *n=72*	**CheckMate 142: Nivolumab arm** *n=74*	**CheckMate 142: Nivolumab/ Ipilimumab arm** *n=119*
**Age** (median [range])	63 [54-71]	53 [44-64]	58 [21-88]
**Metastatic disease diagnosis** (%)			
2002-2005	13 (18)		
2006-2010	14 (19)		
2011-2015	10 (14)	(100) 2014 - 2016	(100) 2015-2016
2016-2018	34 (47)		
Unknown	1 (1)		
**Female** (%)	31 (43)	30 (41)	49 (41)
BRAF (%)			
BRAF wild-type	39 (54)	55 (74)	74 (63)
BRAF mutation	21 (29)	12 (16)	29 (24)
Unknown	12 (17)	7 (9)	15 (13)
**WHO Performance score** (2nd line; %)			
Score 0	11 (15)	32 (43)	54 (45)
Score 1	15 (21)	42 (57)	65 (55)
Unknown	46 (64)		
**Sidedness** (%)			
Right-sided	41 (57)	Unknown	65 (55)
Left-sided	20 (28)		45 (38)
Rectosigmoid/Rectum	9 (12)		6 (5)
Colon NOS	2 (3)		3 (3)
**Stage** (%)			
I	0 (0)	15 (20)	0 (0)
II	5 (7)	0 (0)	14 (12)
III	10 (14)	26 (35)	52 (44)
IV	56 (78)	33 (45)	53 (45)
Unknown	1 (1)		
**Trial participation** (%)	29 (40)	74 (100)	119 (100)
**Treatment lines given** (%)			
0 lines	-	1 (1)	1 (1)
1 line	-	11 (15)	27 (23)
2 lines	51 (71)	22 (30)	43 (36)
≥ 3 lines	21 (29)	40 (54)	48 (40)

Characteristics of the current cohort second-line patients are displayed alongside the baseline characteristics of the CheckMate 142 nivolumab and nivolumab/ipilimumab cohorts.[12, 13] Abbreviations: Colon not otherwise specified (colon NOS), WHO (World Health Organisation).
